# Tube feeding decreases pneumonia rate in patients with severe dementia: comparison between pre- and post-intervention

**DOI:** 10.1186/s12877-017-0662-6

**Published:** 2017-11-21

**Authors:** Shintaro Takenoshita, Keiko Kondo, Keiichi Okazaki, Akihiko Hirao, Keiko Takayama, Keisuke Hirayama, Hiroyuki Asaba, Kenji Nakata, Hideki Ishizu, Hiromi Takahashi, Hanae Nakashima-Yasuda, Yasue Sakurada, Kengo Fujikawa, Osamu Yokota, Norihito Yamada, Seishi Terada, Middle Western Japan-Dementia Study (mid-Dem study)

**Affiliations:** 1 Department of Neuropsychiatry, Okayama University Graduate School of Medicine, Dentistry and Pharmaceutical Sciences, 2-5-1 Shikata-cho, Kita-ku, Okayama, 700-8558 Japan; 2 Department of Psychiatry, Sekizen Hospital, Tsuyama, Japan; 3 Department of Psychiatry, Hayashi Hospital, Okayama, Japan; 4 Department of Psychiatry, Kawada Hospital, Okayama, Japan; 5 Department of Psychiatry, Kibogaoka Hospital, Tsuyama, Japan; 6 Department of Psychiatry, Momonosato Hospital, Kasaoka, Japan; 7 Department of Psychiatry, Kohnan Hospital, Tamano, Japan; 8 Department of Psychiatry, Taiyo Hills Hospital, Takahashi, Japan; 9 Department of Psychiatry, Zikei Hospital, Okayama, Japan; 10 Department of Psychiatry, Mannari Hospital, Okayama, Japan; 11 Department of Psychiatry, Kinoko Espoir Hospital, Kasaoka, Japan

**Keywords:** Dementia, Nasogastric tube, Percutaneous endoscopic gastrostomy, Pneumonia, Tube feeding

## Abstract

**Background:**

It is widely supposed that there is no benefit, including extended survival and decreased rate of pneumonia, in patients with severe dementia receiving enteral tube feeding (TF). However, there have been few studies comparing the frequency of pneumonia before and after TF in severe dementia.

**Methods:**

Nine psychiatric hospitals in Okayama Prefecture participated in this retrospective survey. All inpatients fulfilling the entry criteria were evaluated. All subjects suffered from difficulty in oral intake. Attending physicians thought that the patients could not live without long-term artificial nutrition, and they decided whether or not to make use of long-term artificial nutrition from January 1, 2014 to December 31, 2014.

**Results:**

We evaluated 58 patients including 46 with TF and 12 without. The mean age of all patients was 79.6 ± 9.0 years old. Patients with probable Alzheimer’s disease (*n* = 38) formed the biggest group, and those with vascular dementia the second (*n* = 14). Median survival times were 23 months among patients with TF and two months among patients without TF. The start of TF decreased the frequency of pneumonia and the use of intravenous antibiotics.

**Conclusions:**

TF decreased pneumonia and antibiotic use, even in patients with severe dementia. The results of this study do not necessarily indicate that we should administer TF to patients with severe dementia. We should consider the quality of life of patients carefully before deciding the use or disuse of TF for patients with severe dementia.

## Background

The number of patients with dementia is rising remarkably in Japan. In most patients with severe dementia, difficulties in eating and/or swallowing occur [1], but the decision-making capacity of patients with severe dementia is lost in almost all cases [2]. Therefore, decisions regarding tube feeding (TF) in dementia without an advance directive remain ethically difficult for all people involved [3].

A systematic review evaluating the consequences of TF for patients with severe dementia reported no evidence of extension of survival time in patients receiving enteral TF [4], but the quality of the evidence is mostly poor and relatively old [5]. Recently, another systemic review stated that most studies found no harmful outcome with enteral nutrition use in patients with severe dementia [6], and improvement in nutrition and reduction of inflammation due to TF were reported by a study with long follow-up periods [7].

There have been a few reports dealing with pneumonia in severe dementia patients undergoing TF [8, 9]. The Japanese study revealed that the incidence of aspiration pneumonia in patients with a percutaneous endoscopic gastrostomy (PEG) tube was 9.4% after six months of TF, whereas it was 52.9% in patients with a nasogastric (NG) tube [8]. Among patients with aspiration pneumonia before PEG TF, 51.6% had a recurrence within 6 months after initiation of TF [8]. An Italian study comparing the frequency of aspiration pneumonia between patients with and without dementia found no differences between the two groups [9]. However, there have been no studies comparing the frequency of pneumonia in severe dementia between patients with and without TF. In this study, we examined the frequency of pneumonia before and after TF in severe dementia, and compared the occurrence of pneumonia between severe dementia patients with and without TF.

## Methods

### Design

This study retrospectively compared pre- and post-intervention incidences of pneumonia.

### Setting and subjects

Almost all patients with behavioral and psychological symptoms of dementia (BPSD) are treated in psychiatric hospitals in Japan, and quite a few patients with dementia remain there long-term, even after BPSD are well controlled. Therefore, many dementia patients die in psychiatric hospitals in Japan.

We enlisted the member hospitals of the Association of Okayama Psychiatry Hospitals in a survey of inpatients undergoing artificial feeding. Of 20 psychiatric hospitals in Okayama Prefecture, three do not care for patients with dementia or psychiatric diseases in their chronic or terminal state. Nine of the remaining 17 agreed to participate in this survey.

All patients fulfilled following criteria. (i) They were inpatients in psychiatric hospitals in Okayama Prefecture. (ii) Oral intake was difficult for them. (iii) Attending physicians judged that long-term artificial nutrition was necessary for survival. (iv) The decision on whether or not to make use of long-term artificial nutrition was made by attending physicians between January 1, 2014 and December 31, 2014. (v) Patients suffering from terminal cancer were excluded.

### Artificial nutrition

Artificial hydration and nutrition includes enteral and intravenous nutrition. Enteral nutrition mainly consists of NG and PEG TF, while intravenous nutrition comprises peripheral venous nutrition (PVN) and total parenteral nutrition (TPN). TPN is usually used in the terminal state of malignancy and now rarely used for long-term care at psychiatric hospitals in Japan [10]. Patients receiving TPN were not evaluated in this study. The patients in both groups (TF or PVN) were fed orally before initiation of TF or PVN, and all of them had difficulty in eating orally during the 12 weeks before the decision.

Almost all inpatients in a terminal state in Japan receive artificial nutrition, and this study included no cases in a terminal state receiving both enteral nutrition and intravenous nutrition. Therefore, in this study, all patients not ingesting feeding tube nutrition received PVN in addition to oral intake.

### Clinical diagnosis

All patients with Alzheimer’s disease (AD) were diagnosed according to the criteria for probable AD formulated by the NIA-AA [11]. All patients with vascular dementia (VaD) met the criteria for probable VaD of the AHA-ASA [12]. Other disorders were diagnosed according to ICD-10 criteria.

### Questionnaires

Clinical characteristics of patients including age, sex, clinical diagnosis, methods of artificial nutrition, and duration of artificial nutrition were surveyed. Questionnaires on all subjects were completed by geriatric psychiatrists who knew the patient well and were chiefly in charge of the participants being evaluated. All raters had daily contact with the individuals being studied.

All medical records including nursing records and temperature tables were thoroughly examined by geriatric psychiatrists. They also evaluated the severity of dementia at the time of the decision whether or not to make use of long-term artificial nutrition by using the clinical dementia rating (CDR) [13] and functional assessment staging test (FAST) [14]. Physical comorbidity was evaluated using the Charlson Comorbidity Index (CCI) [15].

In patients receiving TF, records for a maximum 12 weeks before and 12 weeks after the start of TF were considered. The number of days of hospitalization in the psychiatric hospital was counted. The number of days when fever of 38 degrees and over was recorded, the number of days when intravenous antibiotics were used, and the number of bouts pneumonia during the observation period were counted.

### Statistics

Statistical analyses were performed using IBM SPSS Statistics 23.0. Student’s t-test was used to compare two independent groups. Comparisons of proportions between two independent groups were calculated using a chi square test (2 × 2 table). The values for the same patient between before and after the intervention were compared using a paired t-test. The survival time of each group was plotted as a Kaplan-Meier survival curve, and survival times of groups were compared using a log-rank test. The effects of several variables (TF or PVN, age, sex, CCI scores) on survival time were investigated using Cox proportional hazards regression analysis. All *p* values were two-tailed, and *p* < 0.05 was accepted as significant.

## Results

### Comparison of patients with and without TF

This study evaluated 58 patients (31 women and 27 men) (Table 1). The mean age of all patients was 79.6 ± 9.0 years. Clinical diagnoses are shown in Table 1. Of all subjects, patients with probable AD (*n* = 38) formed the biggest group, and those with VaD (*n* = 14) the second. The patients undergoing TF comprised those with a PEG tube (*n* = 20) and those with a NG tube (*n* = 26). All patients were scored as FAST 6e or over (Table 1).

**Table 1:** Comparison of patients with and without tube feeding (*n* = 58)

	With tube	Without tube		
	*n* = 46	*n* = 12	t	*p*
Age (years) (mean ± S.D.)	78.4 ± 8.9	84.0 ± 8.1	1.962	0.055
CCI total	2.2 ± 1.1	2.5 ± 1.4	0.694	0.491
CDR SoB	17.4 ± 1.1	17.4 ± 1.2	0.188	0.851
			χ2	*p*
Sex (men/women)	20/26	7/5	0.844	0.358
Diagnosis				
Alzheimer’s disease	30	8		
Vascular dementia	11	3		
Others	5	1		
FAST				
6e	19	8		
7a	7	1		
7b	12	2		
7c	8	1		
CDR, Personal Care				
2	1	0		
3	45	12		

*S.D.* standard deviation, *CCI* Charlson Comorbidity Index, *CDR* clinical dementia rating, *SoB* sum of boxes

**Fig. 1:**
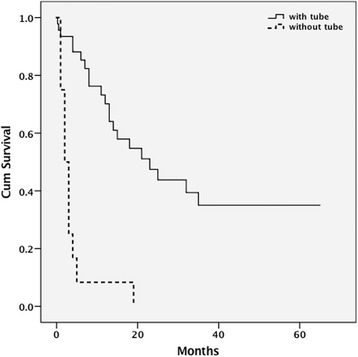
Kaplan-Meier survival curves of patients with and without tube feeding

Survival curves of patients with and without TF are shown in Fig. 1. Patients with TF survived a median of 23 months and patients without TF survived two months. A log-rank test showed significantly longer survival of patients with TF than that of patients without TF (chi-square 33.018, *p* < 0.001). In Cox proportional hazards regression analysis, TF was associated with significantly longer survival (hazard ratio 9.8, 95% confidence interval 3.6–27.0, p < 0.001).

### Comparison between before and after the beginning of TF

Pneumonia occurred more frequently in the 12 weeks before the start of TF than in the 12 weeks after (Table 2). Intravenous antibiotics were used on more days before the start of TF than after (Table 2). In patients without TF, no significant change in the frequency of pneumonia and antibiotic use was seen before and after the decision not to use TF (Table 2).

**Table 2:** Comparison between before and after decision for tube feeding (n = 58)

	12 weeks before	12 weeks after	t	*p*
With tube feeding (n = 46)				
Obsevation days (mean ± S.D.)	70.3 ± 21.7	65.3 ± 24.0	1.124	0.267
Pneumonia (number of bouts)	0.9 ± 0.9	0.4 ± 0.5	4.456	< 0.001
Fever (days)	2.9 ± 4.3	1.9 ± 3.9	1.282	0.206
Antibiotics (days)	11.8 ± 13.2	5.2 ± 7.4	3.308	0.002
Without tube feeding (n = 12)				
Obsevation days (mean ± S.D.)	68.6 ± 17.1	66.6 ± 22.1	0.248	0.809
Pneumonia (number of bouts)	0.6 ± 0.7	0.7 ± 0.8	−0.266	0.795
Fever (days)	1.5 ± 1.2	2.1 ± 3.1	−0.516	0.616
Antibiotics (days)	1.4 ± 3.1	4.1 ± 7.0	−1.104	0.293

*S.D.* standard deviation, *Antibiotics* intravenous antibiotics

Of 18 patients without pneumonia in the observation period before TF, 4 (22%) suffered from pneumonia in the observation period after initiation of TF. Meanwhile, 16 of 28 patients (57%) with pneumonia in the observation period before TF did not suffer from pneumonia in the observation period after initiation of TF.

Comparison of patients with and without TF revealed no significant difference in the number of observation days (before and after decision for TF), bouts of pneumonia (before and after), days of fever (before and after), and days of antibiotic use (after) (Table 2). Days of antibiotic use in the 12 weeks before the decision for TF was higher in patients with TF than in patients without TF (Table 2).

## Discussion

In this study, we evaluated the detailed state of patients with severe dementia at the start of tube nutrition, and found that patients with TF survived longer than those without TF. There have been few studies focusing on the stage of dementia severity when attending physicians decided whether or not to make use of long-term artificial nutrition for patients with severe dementia. Surprisingly, more than 40% of dementia patients started to receive TF at the FAST 6E stage in this study. In some reports, advanced dementia was defined as stage 7A or above on the FAST scale [4, 16]. If the category of FAST 7A or above is used to define advanced dementia, nearly half of dementia patients started to receive TF before they reached the advanced FAST 7A stage. Thus, we think that requiring stage FAST 7A or above for a diagnosis of advanced dementia is too strict.

The level of dementia severity of patients with and without TF was not investigated in most previous studies. The Cochrane review of TF for severe dementia patients includes seven studies [4]. In the study of Peck et al., 52 patients with TF all scored zero on MMSE, but only 71% of 52 patients without TF had dementia and scored <23 on MMSE [17]. In the study of Jaul et al., only 68% of patients with TF and 36% of those without TF were dementia patients. In other studies, only patients with severe dementia were included [18]. However, in those studies, numerous patients who did not need parenteral nutrition were included. It is improbable that patients who needed parenteral nutrition were physically or cognitively equal to those who did not need it. Therefore, there have been no studies in which dementia severity was scored for patients meeting the two following conditions: (i) attending physicians thought that the patients could not live without long-term artificial nutrition, and (ii) attending physicians decided whether or not to make use of long-term artificial nutrition. This study is the first trial to compare dementia patients from two groups with similar severity.

It was previously reported that PEG decreased the frequency of aspiration pneumonia in patients with dementia [8]. However, in that study, dementia severity was not estimated in detail. In this study, we first showed that TF decreased the frequency of pneumonia even in patients with severe dementia. On the other hand, in patients without TF, a decrease in the frequency of pneumonia and use of antibiotics was not observed after the decision not to use TF compared to the frequency before the decision not to use TF.

This study has several limitations. First, detailed laboratory findings at the start of artificial nutrition were not evaluated. Second, we did not collect data on clinical states such as quality of life after initiation of TF. Third, all subjects in this study were inpatients in psychiatric hospitals, and patients in nursing homes were not included. Therefore, we assume that subjects in this study are not representative of all dementia patients. Fourth, this study was not a randomized controlled study. Basically, patients whose conditions were too severe to permit TF were not included. However, it is probable that attending physicians might have tended to unconsciously select PVN for patients whose condition was too severe to benefit from TF. Therefore, the difference in physical severity between the two groups receiving PVN and receiving TF affected the difference in survival times between the two groups.

## Conclusions

Enteral nutrition for patients with dementia prolongs survival and decreases the incidence of pneumonia. We think that this study provides the scientific bases on which an ethical decision should be made. Of course, we should differentiate what is from what should be. TF decreases the frequency of pneumonia even in severe dementia, but it does not mean that we should necessarily use TF for patients with severe dementia.

## Abbreviations

AD: Alzheimer’s disease
BPSD: Behavioral and psychological symptoms of dementia
CCI: Charlson Comorbidity Index
CDR: Clinical dementia rating
FAST: Functional assessment staging test
NG: Nasogastric
PEG: Percutaneous endoscopic gastrostomy
PVN: Peripheral venous nutrition
TF: Tube feeding
TPN: Total parenteral nutrition
VaD: Vascular dementia
